# Books Received

**Published:** 1869-08-15

**Authors:** 


### BOOKS RECEIVED.

A TREATISE ON THE DISEASES OF THE EYE. By J. Soelberg Wells Professor of Ophthalmology in King's College, London; Ophthalmic Surgeon to King's College Hospital; and Assistant Surgeon to the Royal Ophthalmic Hospital, Moorfields. First American edition, with additions. Illustrated with two hundred and sixteen engravings on wood, and six colored plates: together with selections from the test types of Prof. E. Jaeger and Dr. H. Snellen. Philadelphia: Henry C. Lea, 1869. Cloth, $6.50. Leather, $7.50. W. B. Keen & Cooke, Chicago.

The zeal, industry and success of the ophthalmologists are very great, and prove conclusively the advantage to science of devotion to specialties. While, however, we advance this belief, let it be understood that we are, also, firm in our convictions that years of cultivation of the science of medicine as a whole are necessary as the ground-work of any specialty. Specialism thus founded we commend; specialism which springs immediately from a single root is a sickly plant, and should be rooted out completely. Men who have cultivated and practiced the science of medicine as a whole, and whose tastes lead them into special fields are deserving of all confidence; but the little men who have hatched from a *special* egg are not worthy to be entrusted with any interests whatsoever.

In a work now before us we find ophthalmological science brought down to the standard of the present; and we can but notice that in doing this our author expunges as well as adds. For instance, the old operation of reclination or couching is disposed of in half a page, and is introduced by the following sentence: "I mention this operation to state that, in my opinion, it should be completely abandoned." This is stronger language than Stellwag uses, who says, when speaking of the percentage of bad results: "Hence, at present, depression is almost abandoned."

The section upon astigmatism is comprehensive and clear; it is also very finely illustrated, as is, also, the whole work. The whole of chapter XIII on the anomalies of refraction and accommodations of the eye is, to us, the most satisfactory exposition we have yet had.

The same may be said of the chapter devoted to diseases of the lachrymal apparatus.

In section 12 of chapter 1 there is illustrated an ingenious plastic procedure for the cure of simblepharon This difficulty has, in some of its forms, been a stumbling block to surgeons, and if the conjunction admits of such plastic operations as are here described and illustrated, this unfortunate impediment is overcome.

In short, we heartily commend the book to our brethren; not only those who devote themselved to ophthalmology, but to all; for all should know more about the eye than, we regret to say, too many do.

---

A MANUAL OF ELEMENTARY CHEMISTRY, THEORETICAL AND PRACTICAL. By George Fownes, Ph. D. From the Tenth revised and enlarged English edition. With one hundred and ninety-seven illustrations. Edited by ROBERT BRIDGES, M.D., Professor of Chemistry in the Philadelphia College of Pharmacy. In one large and handsome royal 12mo. volume of about 850 closely printed pages: extra cloth, $2.75; leather, $3.25. Philadelphia: Henry C. Lea. Chicago: W. B. Keen & Cooke.

Some years having elapsed since the appearance of the last American edition, and several revisions having been made of the work in England during the interval, it will be found very greatly altered, and enlarged by about one hundred and fifty pages, containing nearly one-half more matter than before. The editors, Mr. Watts and Dr. Bence Jones, have labored sedulously to render it worthy, in all respeets, of the very remarkable favor which it has thus far enjoyed, by incorporating in it all the most recent investigations and discoveries, in so far as is compatible with its design as an elementary text-book. While its distinguishing characteristics have been preserved, various portions have been re-written, and especial pains have been taken with the department of Organic chemistry, in which late researches have accumulated so many new facts, and have enabled the subject to be systematized and rendered intelligible in a manner formerly impossible. As only a few months have elapsed since the work thus passed through the hands of the English editors, but little has remained to be done by the American editor. Such additions as seemed advisable have, however, been made, and especial care has been taken to secure, by the closest scrutiny, the the accuracy so essential in a work of this nature.

The amount of matter condensed into this small volume is almost wonderful. It is pre-eminently the text-book for the student and practitioner, and will, of course, achieve a large success and wide popularity.

---

HOW TO READ CHARACTER: A New Illustrated Hand-Book of Phrenology and Physiognomy, for the use of Students and Examiners; with a Descriptive Chart for marking, and upwards of 170 Engravings. Price. post-paid, in muslin, $1.25; in paper, $1. S. R. Wells, Publisher, 389 Broadway, New York. W. B. Keen & Cooke.

---

A TREATISE ON THE FUNCTION OF DIGESTION: Its Disorders and their Treatment. By F. W. PAVY, M.D., F. R. S., etc., etc. From the Second London Edition. Pp. 246. Philadelphia; Henry C. Lea. 1869. $3.00. Chicago: W. B. Keen & Cooke.

The author sustains in this brief *resume* his high reputation. His monograph is a model of condensation, yet a perspicuous view of a most important subject. It is aphoristic, yet eminently practical—a worthy companoin to Chamber's excellent treatise.

### PAMPHLETS.

BRAITHWAITE'S RETROSPECT OF PRACTICAL MEDICINE AND SURGERY. Part LIX, July, 1869. New York: W. A. TOWNSHEND & ADAMS Publishers. $2.50 a year in advance, postage prepaid. Half-yearly parts, $1.50.

---

PROCEEDINGS OF THE STATE MEDICAL SOCIETY OF MICHIGAN FOR 1867 AND 1868.

MONTHLY REPORT OF THE DEPARTMENT OF AGRICULTURE for May and June, 1869.

---

GEORGIA MEDICAL ASSOCIATION. Proceedings and Transactions at the Sessions of 1868 and 1869, with Constitution and By-Laws.

---

UNANIMOUS REPORT OF SELECT COMMITTEE ON REPRESENTATIVE REFORM. Presented to the Senate of the United States, March 2, 1869, by Hon B. F. WADE, Senator from Ohio. Reprinted and electrotyped by the Minority Representation Society, Chicago, Ill.

Copies of this valuable paper can be obtained by addressing the Secretary of the Society, Sidney Myers, Esq., Chicago. It would be out of place here to express our earnest hope that the important subject of which it treats may be thoroughly studied by the Medical Profession, who should be first and foremost in every grand reform.

---

PRESCRIPTION AND CLINIC RECORD. By EDOUARD SEGUIN, M.D. Published by W. Wood, 61 Walker Street, New York. Fifth edition, Price 50 cts.

A convenient pocket arrangement whereby duplicates of prescriptions may be readily kept, as also brief notes of each case. An introductory chapter affords some valuable hints.

---

TREATMENT OF LACHRYMAL AFFECTIONS. By PROF. ARLT, of the University of Vienna. Translated by JOHN F. WEIGHTMAN, M.D. Philadelphia. Lindsay & Blakiston, 1869. Pp. 30. With Plates. A very useful monograph.

---

STRUCTURAL LESIONS OF THE SKIN: Their Pathology and Treatment, Illustrated. By HOWARD DAMON, A.M., M.D., Fellow of the Mass. Med. Soc., etc., etc. Philadelphia, J. B. Lippincott & Co. 1869. W. B. Keen & Cooke, Chicago.

---

ANNUAL ANNOUNCEMENTS, Bellevue Hospital Medical College, 1869-70; Med. Dept. University of Buffalo; Chicago Medical College; Missouri Medical College.

---

SECOND ANNUAL REPORT OF THE BRAINARD FREE DISPENSARY OF THE CITY OF CHICAGO.

---

DEMANDS OF THE HOUR UPON THE MEDICAL STUDENT. Valedictory Address, by L. M. WHITING, M.D., Charity Hospital Med. College, Cleveland, Ohio.

---

THE RELATIONS AND RECIPROCAL OBLIGATIONS BETWEEN THE MEDICAL PROFESSION AND THE EDUCATED AND CULTIVATED CLASSES. By HENRY S. HEWITT, M.D. Being an Oration before the Alumni Association of the Medical Department of the University of New York.

